# Deacetylation via SIRT2 prevents keratin-mutation-associated injury and keratin aggregation

**DOI:** 10.1172/jci.insight.166314

**Published:** 2023-07-24

**Authors:** Jingyuan Sun, Pei Li, Honglian Gui, Laure Rittié, David B. Lombard, Katrin Rietscher, Thomas M. Magin, Qing Xie, Li Liu, M. Bishr Omary

**Affiliations:** 1 Center for Advanced Biotechnology and Medicine, Rutgers University, Piscataway, New Jersey, USA.; 2 Department of Radiation Oncology, Nanfang Hospital, Southern Medical University, Guangzhou, PR China.; 3 Department of Infectious Diseases, Ruijin Hospital, Jiaotong University School of Medicine, Shanghai, PR China.; 4 Department of Dermatology, University of Michigan, Ann Arbor, Michigan, USA.; 5 Sylvester Comprehensive Cancer Center, and Department of Pathology & Laboratory Medicine, University of Miami Miller School of Medicine, Miami, Florida, USA.; 6 Division of Cell and Developmental Biology, Institute of Biology, Leipzig University, Leipzig, Germany.; 7 Hepatology Unit and Department of Infectious Diseases, Nanfang Hospital, Southern Medical University, Guangzhou, PR China.; 8 Department of Molecular & Integrative Physiology, University of Michigan, Ann Arbor, Michigan, USA.

**Keywords:** Dermatology, Hepatology, Apoptosis, Cytoskeleton, Drug screens

## Abstract

Keratin (K) and other intermediate filament (IF) protein mutations at conserved arginines disrupt keratin filaments into aggregates and cause human epidermolysis bullosa simplex (EBS; K14-R125C) or predispose to mouse liver injury (K18-R90C). The challenge for more than 70 IF-associated diseases is the lack of clinically utilized IF-targeted therapies. We used high-throughput drug screening to identify compounds that normalized mutation-triggered keratin filament disruption. Parthenolide, a plant sesquiterpene lactone, dramatically reversed keratin filament disruption and protected cells and mice expressing K18-R90C from apoptosis. K18-R90C became hyperacetylated compared with K18-WT and treatment with parthenolide normalized K18 acetylation. Parthenolide upregulated the NAD-dependent SIRT2, and increased SIRT2-keratin association. SIRT2 knockdown or pharmacologic inhibition blocked the parthenolide effect, while site-specific Lys-to-Arg mutation of keratin acetylation sites normalized K18-R90C filaments. Treatment of K18-R90C–expressing cells and mice with nicotinamide mononucleotide had a parthenolide-like protective effect. In 2 human K18 variants that associate with human fatal drug-induced liver injury, parthenolide protected K18-D89H– but not K8-K393R–induced filament disruption and cell death. Importantly, parthenolide normalized K14-R125C–mediated filament disruption in keratinocytes and inhibited dispase-triggered keratinocyte sheet fragmentation and Fas-mediated apoptosis. Therefore, keratin acetylation may provide a novel therapeutic target for some keratin-associated diseases.

## Graphical abstract

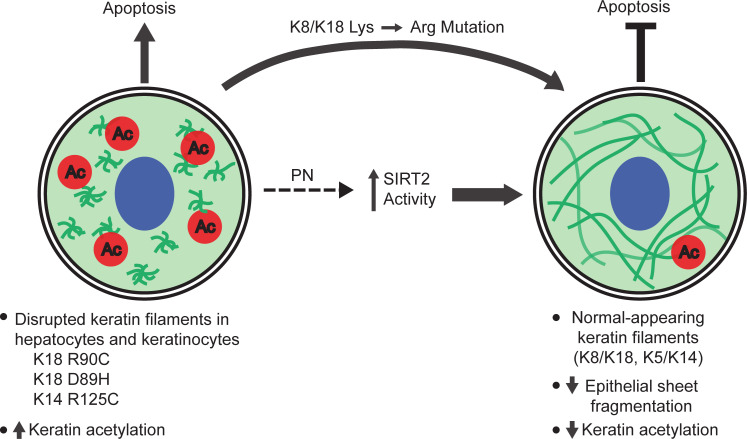

## Introduction

Intermediate filaments (IFs) make up a large family of cytoplasmic and nuclear cytoskeletal proteins that are expressed in a cell-selective manner and whose mutation accounts for more than 70 IF-associated diseases (IF-pathies) (1–5). Among IFs, keratins make up the largest family, with 54 functional keratin genes (6). Keratins include type I (K9-K28, K31-K40) and type II (K1-K8, K71-K86) proteins, which are expressed as obligate noncovalent heteropolymer pairs in an epithelial cell–specific manner (7) (e.g., K8/K18 in simple-type epithelia, K5/K14 in basal keratinocytes). A common structural feature among all IFs is a conserved central α-helical rod domain that is flanked by less conserved N-terminal head and C-terminal tail domains (1, 7). Among IFs, R90 of K18 is a highly conserved residue, and its mutation in epidermal keratins (K14-R125C) was the first link of an IF mutation to any human disease (in this case, epidermolysis bullosa simplex [EBS]; see refs. 8, 9). The K18-R90C mutation, when expressed in cultured cells or as a transgene in mice, causes the collapse of liver keratin filaments into dots and aggregates, and leads to keratin hyperphosphorylation and predisposition to Fas-induced and several other types of liver injury (10–12). Similarly, mutations at this highly conserved residue and other conserved residues (e.g., K18-D89H and K8-K339R whose mutation associates with human fatal drug-induced liver injury) lead to disruption of the filamentous keratin cytoskeleton into aggregates and consequent cell and tissue fragility (8–10, 13–15).

Given the lack of directed therapy toward the diverse IF-associated diseases, we undertook an unbiased high-throughput drug-screening approach using A549 human lung adenocarcinoma cells that were transduced with green fluorescent protein (GFP)-K18-R90C. High-throughput drug screening has been utilized to identify compounds that stabilized IFs and potentially serve as direct or indirect chemical chaperones (16). This approach has successfully identified the pan-kinase inhibitor PKC412 as a compound that reverts disrupted K18-R90C aggregates into a WT-like extended filament network by enhancing keratin association with nonmuscle myosin heavy chain IIA (NM-IIA) in a myosin-dephosphorylation-regulated manner (17), or in patient-derived keratinocytes expressing K14-R125C (18). Also, by using this high-throughput drug screening system, another compound termed PP2, a SRC-family tyrosine kinase inhibitor, was identified and demonstrated to protect cells from keratin-mutation-associated liver injury and filament disruption via SRC kinase inhibition (19). Our hypothesis is that compounds that convert the mutant keratin phenotype from dots/aggregates to filaments will protect cells and tissues from injury. The power of this approach is that a reverse mechanistic approach can be utilized to identify keratin regulatory mechanisms.

Posttranslational modifications (PTMs) play an important role in regulating the assembly and disassembly dynamics of IF proteins, as well as their associations with other cellular components (20–22). Several stress-induced PTMs, including phosphorylation, transamidation, and sumoylation, have been well studied (23–25). Another keratin regulatory modification is acetylation, as exemplified by acetylation at the K8 conserved residue Lys207, which regulates keratin solubility and filament organization (26). Several other K8/K18 acetylation sites have been identified using high-resolution mass spectrometry (27, 28), but the function of IF acetylation and its crosstalk with other IF PTMs remain poorly understood (22).

In the present study, we used high-throughput dug screening to identify the plant derivative parthenolide (PN) as a compound that potently normalizes K18-R90C–mediated keratin filament aggregation in cells and mouse liver, which in turn protected cells and mice from Fas ligand–induced (Fas-L–induced) apoptosis. Moreover, we demonstrate that PN treatment results in deacetylation of K8/K18 via increased binding with NAD-dependent sirtuin 2 (SIRT2). The importance of keratin deacetylation was supported by pharmacologic inhibition or molecular knockdown of SIRT2, site-specific mutation of keratin acetylation sites in the context of K18-R90C, and treatment of K18-R90C–expressing cells and mice with nicotinamide mononucleotide (NMN). Importantly, the effectiveness of PN extended to the natural variants K18-D89H and K14-R125C. Our findings provide multiple lines of evidence that keratin acetylation provides a potential therapeutic target for some of the IF-pathies by normalizing keratin-mutation-induced filament disorganization.

## Results

### PN treatment normalizes K18-R90C filament disruption and protects against Fas-induced apoptosis in cultured cells and mice.

**Figure 1:**
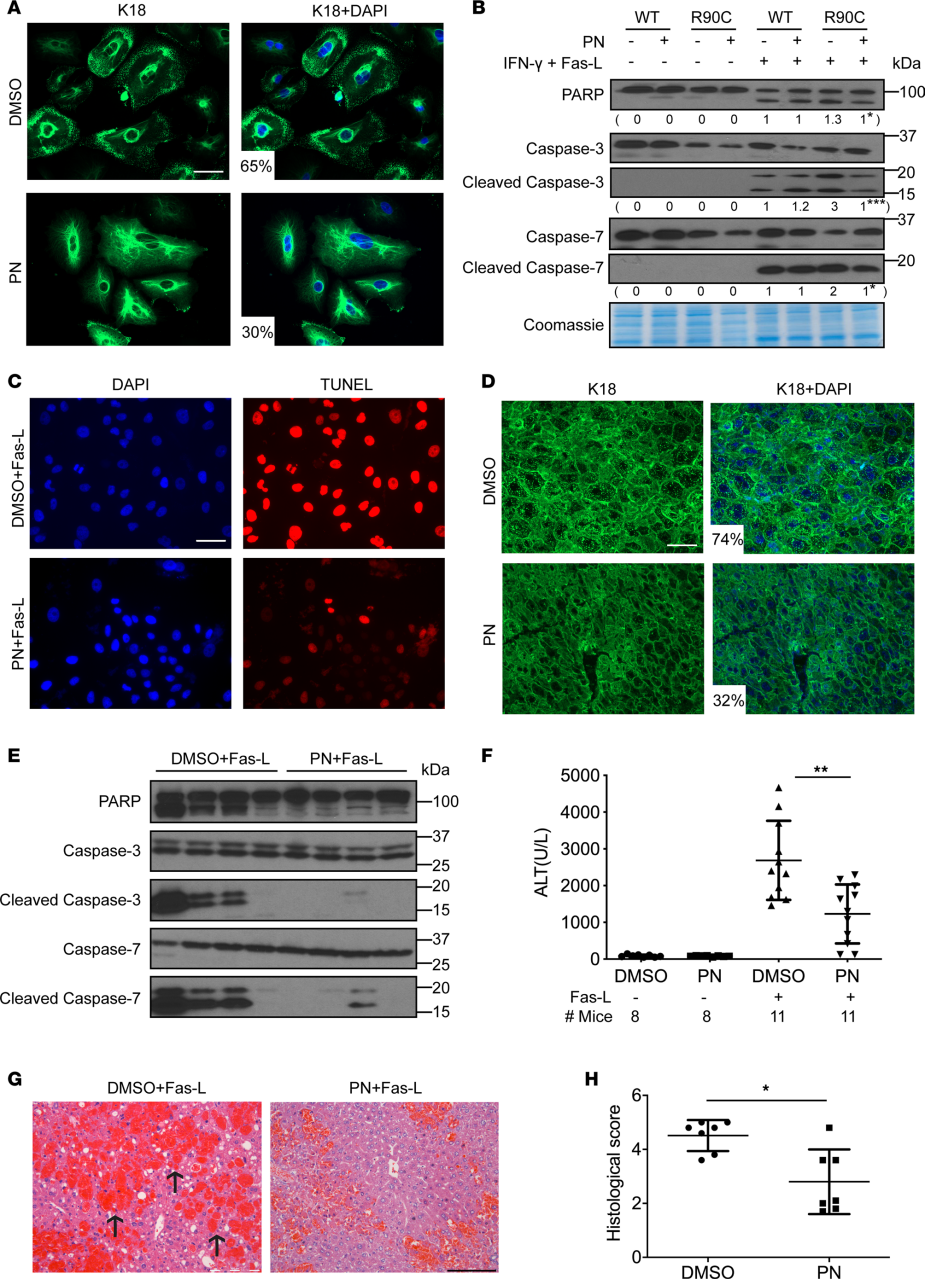
Parthenolide (PN) normalizes K18-R90C filament disruption and protects against Fas-induced apoptosis in cultured cells and mice. (**A**) A549 cells were transduced with lentivirus expressing GFP-tagged K18-R90C and K8-WT (cotransfection with type I and II keratins is needed for physiologic heteropolymer filament formation). After 2 days, cells were treated with DMSO or PN (5 μM, 48 hours) and then fixed and stained with DAPI. Average percentage of cells with dots/group for DMSO vs. PN is shown. *P* < 0.001, from 3 independent experiments). Scale bar: 50 μm. (**B**) GFP-K18-WT and GFP-K18-R90C lentiviruses were transduced into A549 cells followed by treatment with DMSO or PN (5 μM, 48 hours) and then apoptosis was induced using IFN-γ and Fas ligand (Fas-L). Cell lysates were analyzed by blotting using antibodies against the indicated antigens (Coomassie stain shows equal loading). The average relative intensity of the indicated bands from 3 individual experiments is included below each blot. *P < 0.05, ***P < 0.001 using unpaired, 2-tailed Student’s *t* test (comparing DMSO with PN). (**C**) Representative TUNEL staining of GFP-K18-R90C–transduced A549 cells treated with DMSO or PN and then challenged with IFN-γ plus Fas-L. The percentage of TUNEL^+^ cells in DMSO vs. PN was 82% vs. 33%, respectively (*P* < 0.001). Scale bar: 50 μm. For panels **A**–**C**, all experiments were repeated at least 3 times. *P < 0.05, **P < 0.005 using unpaired, 2-tailed Student’s *t* test. (**D**) Transgenic mice that express K18-R90C were treated daily with DMSO or PN (1 mg/kg mouse weight; i.p.) for 4 days. Liver sections were double stained with anti-K18 antibody and DAPI. The percentage of cells with dots/group is shown in Supplemental Figure 1F (*P* < 0.001). Scale bar: 50 μm. (**E**) Mice were treated with DMSO or PN as in panel **D** and then challenged with Fas-L. Livers were collected after 5 hours and analyzed by immunoblotting (each lane represents a different liver). (**F**) Alanine transaminase (ALT) was measured using sera collected from the indicated number of mice. **P = 0.002 using 1-way ANOVA followed by Tukey’s post hoc test. (**G**) Representative images of hematoxylin and eosin–stained liver sections. Arrows highlight areas of hemorrhage. Scale bar: 100 μm. (**H**) Quantification of the hemorrhage after Fas-L treatment of the mice is included. *P = 0.013 using unpaired, 2-tailed Student’s *t* test. For each group (DMSO or PN), 10 sections for each liver and 7 independent livers were used.

We used lentivirally transduced A549 cells that express GFP-K18-R90C as a system to screen for compounds that normalize mutation-induced keratin filament disruption. Compounds of interest were those that decreased the number of keratin dots (i.e., keratin aggregation) per cell, as assessed by fluorescence imaging. Based on a screening of a 1,037-compound library, PN was selected for further characterization since it is a well-studied natural product (29). After treating K18-R90C cells with PN or DMSO as control vehicle, PN had a dramatic effect in normalizing the disrupted K18-containing filaments by decreasing the percentage of cells with dots from 65% to 30% (Figure 1A and Supplemental Figure 1A; supplemental material available online with this article; https://doi.org/10.1172/jci.insight.166314DS1), without altering K8/K18 or other keratin levels (Supplemental Figure 1B). Compared with PKC412, which also reverts disrupted K18-R90C filaments into WT-like extended filaments by enhancing keratin-myosin association in a myosin-dephosphorylation-regulated fashion (17), PN showed earlier significant effects (Supplemental Figure 1, A and C). We also tested dimethylaminoparthenolide (DMAPT), a soluble analog of PN on K18-R90C cells. DMAPT showed a similar corrective effect to that of PN on R90C-induced filament disorganization (Supplemental Figure 1D). One of the major potential benefits of identifying keratin filament–normalizing drugs is to find compounds that can overcome the R90C-induced predisposition to cell injury. Importantly, PN protected A549 cells that express K18-R90C but not K18-WT from apoptosis (induced by IFN-γ and Fas-L), as determined by the decreased level of the apoptosis markers, cleaved PARP and caspases 3 and 7 (Figure 1B). TUNEL assay showed markedly less positive nuclear staining after PN treatment (Figure 1C and Supplemental Figure 1E), further supporting the conclusion that PN protects from apoptosis in K18-R90C–expressing cells.

We then tested the effectiveness of PN in vivo. Administration of PN to transgenic mice that express human K18-R90C led to keratin filament normalization in the livers, with decreased abnormal dot-pattern keratin staining from 74% to 32% (Figure 1D and Supplemental Figure 1F), without altering K8/K18 levels (Supplemental Figure 1G). Similar results were seen in livers of the mutant mice fed DMAPT by gavage (Supplemental Figure 1H). Importantly, PN protected K18-R90C–expressing mice from Fas-L–induced liver injury, as evidenced by significantly decreased apoptosis markers in liver lysates (Figure 1E), lower serum ALT levels (Figure 1F), and improved histopathological features (Figure 1, G and H). However, the PN-mediated protection depends on the R90C mutant context, since there is no protection from Fas-mediated liver injury in nontransgenic mice, K18-null, or K18-WT–overexpressing mice (Supplemental Figure 1, I–K; all in an FVB/N genetic background).

### PN decreases K18 acetylation via SIRT2 in cells and mice.

**Figure 2:**
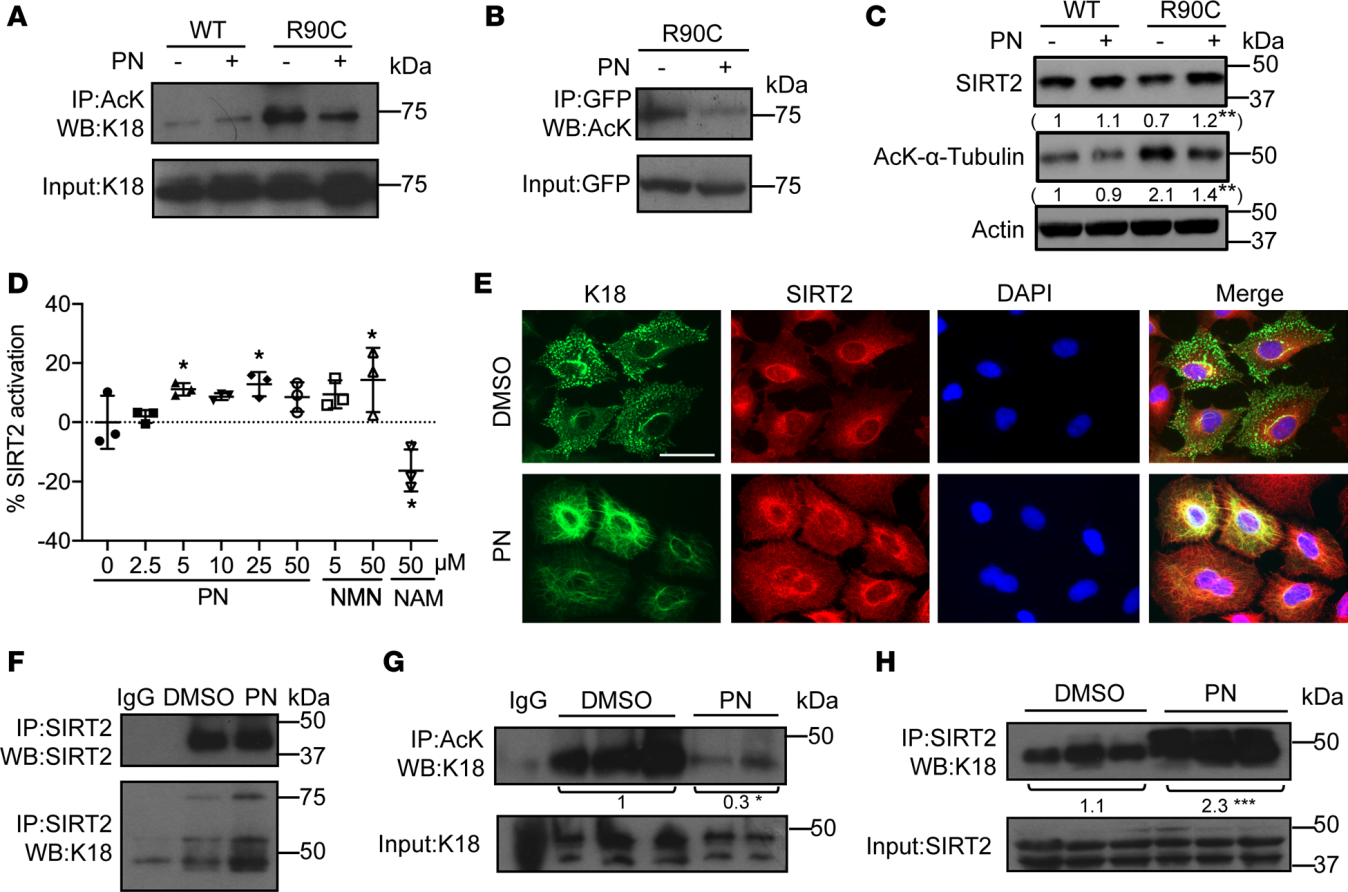
PN decreases K18 acetylation via SIRT2 in cultured cells and mouse liver. (**A**) GFP-K18-WT and GFP-K18-R90C lentivirus–transduced A549 cells were treated with DMSO or PN (5 μM, 48 hours). Acetylated proteins were immunoprecipitated using an anti-AcK antibody followed by blotting with an anti-K18 antibody. (**B**) GFP-K18-R90C lentivirus–transduced A549 cells were used for immunoprecipitation with an anti-GFP antibody then blotted with an anti-AcK antibody. An input blot is also shown. (**C**) Lysates from GFP-K18-WT/GFP-K18-R90C–transduced cells were blotted with antibodies against SIRT2 or acetylated α-tubulin (actin blot is included as loading control). The average relative intensity of the indicated bands from 3 separate experiments is included beneath each blot. *P < 0.05 using unpaired, 2-tailed Student’s *t* test (comparing R90C DMSO with PN). (**D**) The percentage activation of SIRT2 in the presence of PN, NMN (activator control), and NAM (inhibitor control). *P < 0.05 using the least significant difference test, compared with PN (= 0). (**E**) Immunofluorescent double staining of K18 and SIRT2 in A549 cells expressing K18-R90C treated with DMSO/PN. Scale bar: 50 μm. (**F**) Anti-SIRT2 (and control nonimmune IgG) immunoprecipitates were prepared from cells used in panel **E**, and then blotted with anti-SIRT2/anti-K18 antibodies. (**G**) Liver lysates from DMSO/PN-treated mice expressing K18-R90C were used for immunoprecipitation with an anti-AcK antibody (separate livers/lane) followed by immunoblotting with an anti-K18 antibody (input lysate was similarly blotted). *n* = 3 (DMSO group), *n* = 2 (PN group). (**H**) SIRT2 immunoprecipitates were prepared from liver extracts isolated from K18-R90C–expressing mice pretreated with DMSO/PN. K18 was analyzed by immunoblotting. Input lysates were also blotted with anti-SIRT2 (separate livers/lane are shown). *n* = 3 (DMSO group), *n* = 3 (PN). For panels **G** and **H**, the mean relative band intensity for each of the 2 groups is included beneath each blot. *P < 0.05, ***P < 0.001 using unpaired, 2-tailed Student’s *t* test (comparing DMSO with PN).

We explored the mechanism underlying PN’s effect. Previously, PN was reported to promote HDAC1 depletion and cell death (29), and to lead to regression of cholestasis-induced fibrosis by inhibiting HDAC4 (30). Given our prior characterization of K8 acetylation at Lys-207 and its role in decreasing keratin solubility (26), we examined the acetylation of K18 by performing anti-AcK immunoprecipitation followed by blotting for bound keratins. Notably, K18-R90C was highly acetylated compared with K18-WT, and PN treatment led to a prominent decrease in K18-R90C acetylation (Figure 2A). This deacetylation was supported by carrying out the reciprocal experiment (i.e., anti-GFP immunoprecipitation followed by anti-Ack blot; Figure 2B). We then tested whether the major cytoplasmic deacetylases, SIRT2 and HDAC6, are involved in K18-R90C deacetylation.

Transfection of FLAG-tagged SIRT2 or HDAC6 in BHK or A549 cells showed that SIRT2, but not HDAC6, decreased K18 acetylation (Supplemental Figure 2A). Furthermore, K18-R90C–expressing cells had lower levels of SIRT2 (compared with WT-expressing cells) that increased after PN treatment, commensurate with a decrease in SIRT2 substrate (α-tubulin) acetylation (Figure 2C). To explore whether PN could regulate other sirtuin family members, we tested the mRNA levels of *SIRT1*–*SIRT7* in WT and R90C cells upon PN treatment. The results revealed that PN upregulated the mRNA levels of *SIRT2* and *SIRT5* (Supplemental Figure 2, B–H). However, PN did not alter the protein level of SIRT5 (Supplemental Figure 2I). Thus, we focused on SIRT2 in all subsequent experiments. To test whether PN can activate SIRT2 directly, we performed an in vitro fluorescence-based assay, which showed that PN increases SIRT2 activity at concentrations of 5 μM or higher (Figure 2D). Moreover, keratin-SIRT2 colocalization increased after PN treatment of K18-R90C cells, as determined by immunofluorescent staining and coimmunoprecipitation (Figure 2, E and F, and Supplemental Figure 2J).

We then assessed the distribution of SIRT2 and K18 in K18-R90C livers in the presence or absence of PN, by sequential fractionation with a detergent-free buffer, followed by nonionic (NP-40) and zwitterionic (Empigen) detergent solubilization and compared these fractions to the remaining pellet. As shown in Supplemental Figure 3A, SIRT2 and K18 become more soluble upon cell exposure to PN (e.g., K18 moves from the pellet to the Empigen fraction after PN treatment, and SIRT2 levels decrease in the pellet fraction and are more readily detectable in the hypotonic fraction). In addition, K18 and α-tubulin acetylation in livers of K18-R90C mice was downregulated after 4 daily injections of PN (Figure 2G and Supplemental Figure 3B). Moreover, the association between human K18 and SIRT2 increased prominently, as demonstrated by SIRT2 immunoprecipitation followed by blotting for K18 (Figure 2H).

### Inhibition of SIRT2 impairs the ability of PN to ameliorate K18-R90C aggregation.

**Figure 3:**
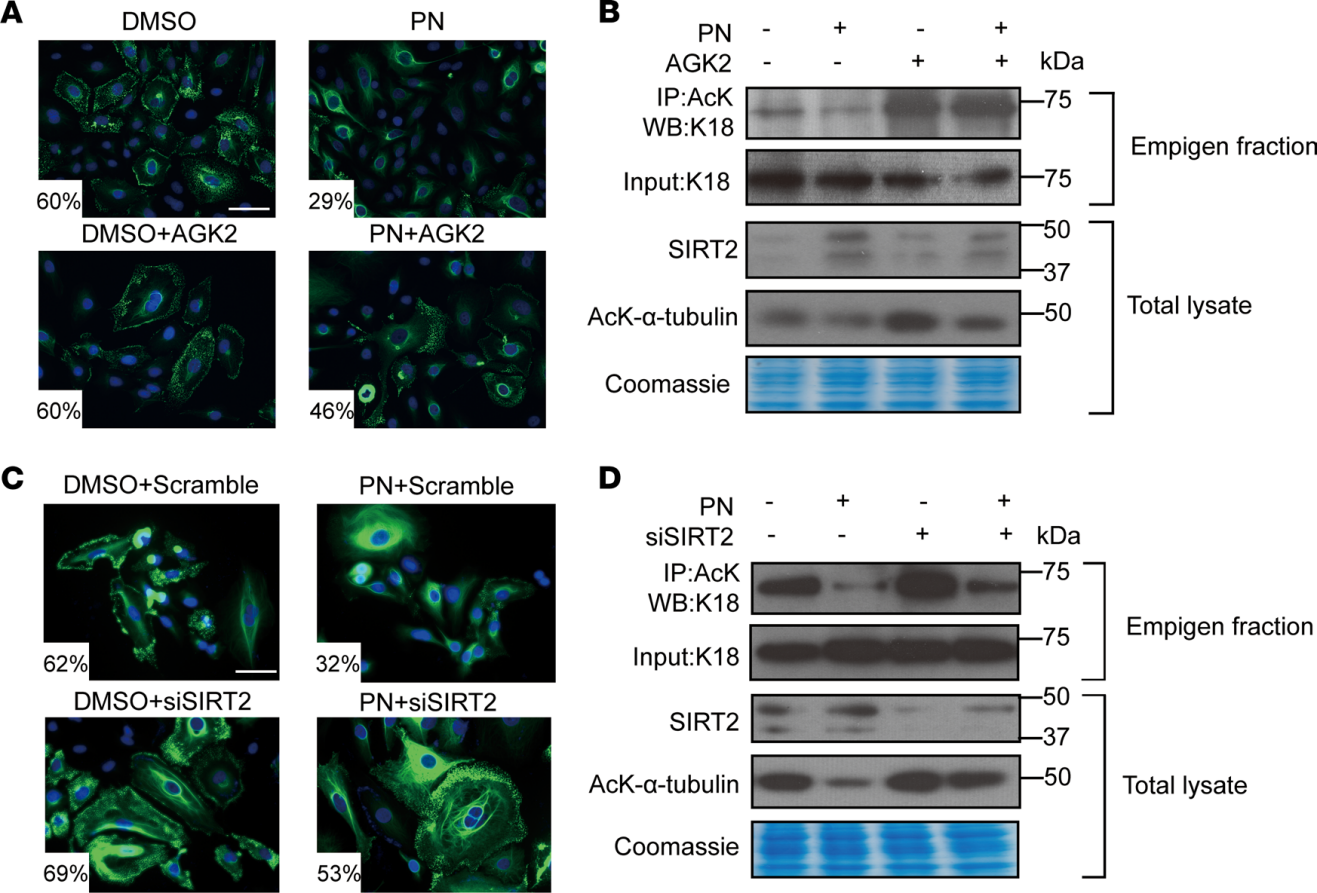
Pharmacological inhibition or knockdown of SIRT2 impairs the ability of PN to ameliorate K18-R90C aggregation. (**A**) K18-R90C–transduced cells were treated with the SIRT2 inhibitor AGK2 (10 μM, ±PN, 48 hours). Images from the stained cells were quantified for the percentage cells with dots. *P* = 0.004, comparing DMSO vs. PN; *P* < 0.05, comparing PN vs. PN + AGK2. (**B**) Duplicate cells from panel **A** were then solubilized with Empigen and used for immunoprecipitation with an anti-AcK antibody followed by blotting of the precipitates/input lysate with anti-K18 antibody. Total lysates from duplicate cells were also blotted with the indicated antibodies. (**C**) K18-R90C–expressing cells were cultured with control or SIRT2 siRNA (24 hours) and then treated with DMSO/PN (48 hours). Images from stained cells were quantified for the percentage cells with dots. (**D**) Duplicate cells from panel **C** were used for immunoprecipitation and blotting as in panel **B**. Coomassie staining is included to show equal loading. Scale bars: 50 μm (**A** and **C**). All experiments were repeated at least 3 times with similar results. One-way ANOVA was used for comparison between treatment groups, and Tukey’s post hoc test was used for 2-group comparisons.

The importance of SIRT2-mediated deacetylation of K18 in normalizing mutant keratin filament disruption was further supported by treating K18-R90C cells with the SIRT2-selective inhibitor AGK2 in the presence or absence of PN. The GFP-K18 fluorescence showed that AGK2 blocked PN’s effect of correcting the R90C-induced filament disruption and increased K18 acetylation (Figure 3A and Supplemental Figure 4A). Similarly, AGK2 treatment inhibited PN’s activation of SIRT2, as determined by α-tubulin acetylation (Figure 3B). Similar findings regarding keratin acetylation and filament organization were observed using siRNA-mediated SIRT2 knockdown (Figure 3, C and D, and Supplemental Figure 4B). We also found that SIRT5 knockdown did not impair PN’s effect on K18-R90C–induced filament disorganization and K18 deacetylation (Supplemental Figure 4, C and D, respectively).

### NMN normalizes K18-R90C–induced keratin filament disruption.

**Figure 4:**
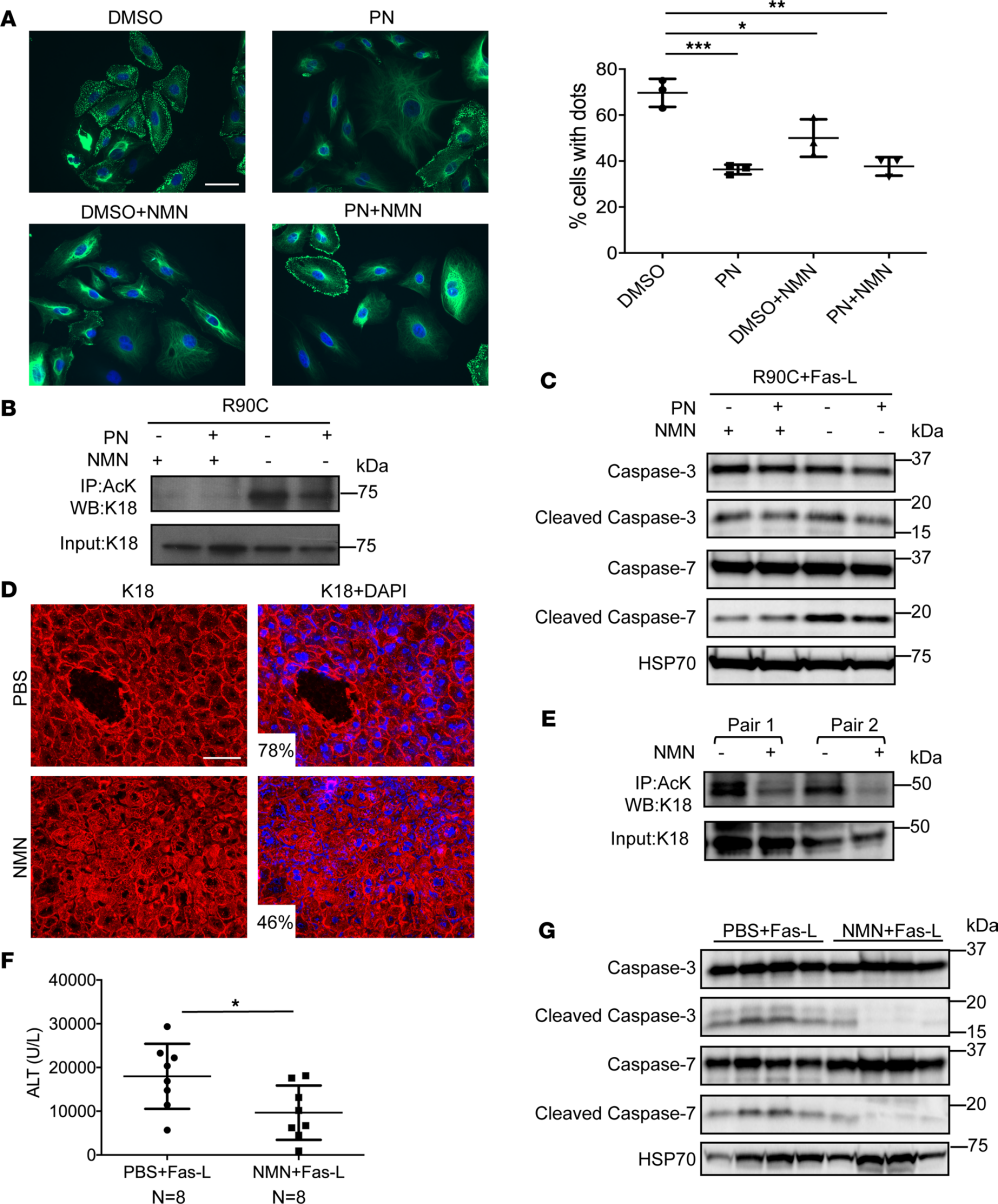
Keratin deacetylation by nicotinamide mononucleotide (NMN) normalizes K18-R90C–induced keratin filament disruption and protects against Fas-induced apoptosis in cells and mouse liver. (**A**) A549 cells were transduced with GFP-K18-R90C followed by treatment with NMN (2 mM), PN (5 μM) or NMN + PN. Cells were imaged after counterstaining the nuclei (blue). Scale bar: 50 μm. Quantification of the percentage cells with dots was done using pooled counts from 3 experiments. Data are presented as mean ± SD. *P = 0.03, **P < 0.01, ***P < 0.001 using 1-way ANOVA followed by Tukey’s post hoc test. (**B**) Lysates from cells similar to those used in panel **A** were used for immunoprecipitation with an anti-AcK antibody followed by immunoblotting with an anti-K18 antibody (the input lysate was similarly blotted). (**C**) Cells as in panel **A** were treated with IFN-γ and Fas-L to induce apoptosis. Cell lysates were analyzed by blotting using antibodies against the indicated antigens. (**D**) Mice that overexpress K18-R90C were treated daily with PBS or NMN for 4 days. Liver sections were then double stained with anti-K18 antibody (red) and DAPI (blue). *P* = 0.01 when comparing the percentage cells with dots in hepatocytes from PBS vs. NMN treatment groups. (**E**) Livers from 2 pairs of K18-R90C mouse siblings were treated with NMN or PBS (4 days) followed by solubilization, AcK immunoprecipitation, and then blotting (of precipitates and input) with an anti-K18 antibody. (**F**) Mice were challenged with Fas-L to induce liver injury, followed by measurement of serum alanine transaminase (ALT). *P < 0.03 using an unpaired, 2-tailed Student’s *t* test. (**G**) Livers from mice used in panel **E** were homogenized followed by blotting with antibodies against the indicated antigens. HSP70 is included as a loading control. Each lane represents livers from separate animals.

In order to further support the importance of (the NAD-dependent, ref. 31) SIRT2 in keratin filament normalization, we examined the effect of NMN in cells and mice that express K18-R90C. There are 4 pathways to synthesize NAD+, including the salvage pathway that utilizes nicotinamide (NAM), nicotinic acid (NA), nicotinamide riboside (NR), or the de novo pathway that uses tryptophan (32). Nicotinamide phosphoribosyltransferase (NAMPT), the rate-limiting enzyme in the NAD+ biosynthetic pathway, produces NMN in 1 step, a key NAD+ intermediate. In many studies, administration of NMN enhances NAD+ biosynthesis in vitro and in vivo, thereby rendering NMN a key precursor with possible therapeutic implications (33–36). This led us to test the effect of NMN on K18-R90C–mediated keratin filament disruption in A549 cells. Strikingly, NMN ameliorated mutant keratin filament aggregation commensurate with decreased K18 acetylation and protected the cells from Fas-induced apoptosis without having an additive effect with PN (Figure 4, A–C). In vivo, NMN improved liver keratin filament organization, together with decreased K18 acetylation and, importantly, protected the mice from Fas-induced liver injury (Figure 4, D–G).

### Keratin deacetylation by lysine point mutation normalizes K18-R90C–induced keratin filament disruption.

**Figure 5:**
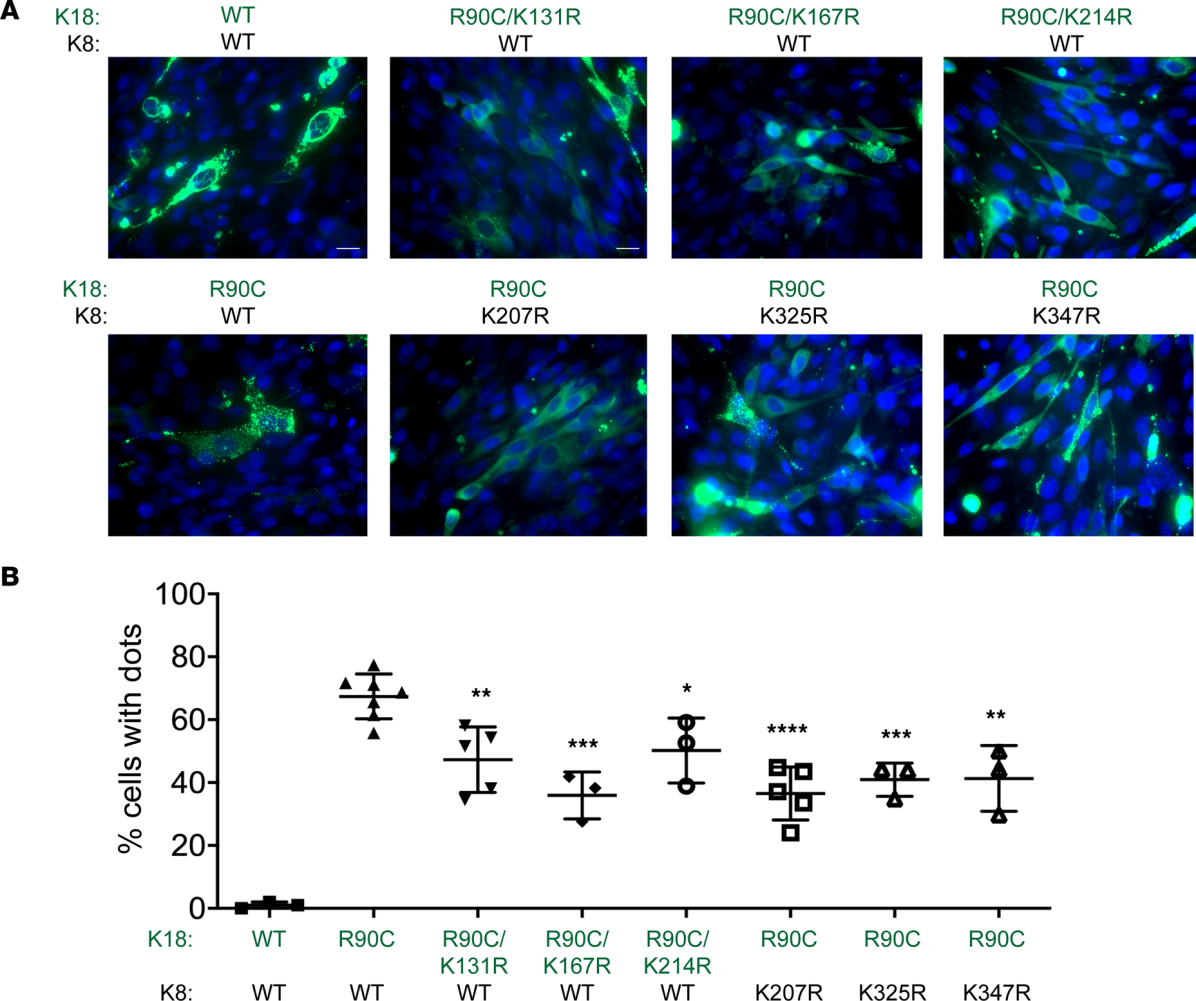
Keratin deacetylation by lysine point mutation normalizes K18-R90C–induced keratin filament disruption. (**A**) Double mutants of K18 (R90C/acetylation) and K18-R90C/K8 acetylation mutants, WT K8 and WT K18, or WT K8 and K18-R90C were cotransfected into NIH-3T3 cells (which do not express endogenous keratins) followed by double staining (green = keratins, blue = nuclei). Scale bars: 50 μm. (**B**) Quantification of the percentage cells with dots using pooled counts from 3 separate experiments. Data are presented as the mean ± SD. *P < 0.05, **P < 0.01, ***P < 0.001, ****P < 0.0001 when comparing each of the double mutant constructs with cells transfected with WT K8 and K18-R90C. One-way ANOVA was used for comparison between treatment groups, and Tukey’s post hoc test was used for comparing multiple groups with the R90C/WT group.

Choudhary et al. identified 3,600 lysine acetylation sites in 1,750 acetylated proteins, with K8/K18 being among these acetylated targets with all sites being in the α-helical rod domain (28). Since K18-R90C was hyperacetylated compared with K18-WT, and PN deacetylated K18-R90C, we selected the 6 previously described K8 Lys-207, Lys-325 and Lys-347; and K18 Lys-131, Lys-167 and Lys-214 acetylation sites for further study. Lysine-to-arginine mutants were generated using K8-WT or K18-R90C followed by transfection in NIH-3T3 cells and assessment of the keratin filament organization. Notably, Lys-to-Arg mutation of K18-R90C at Lys-131/Lys-167/Lys-214, or K8-WT at Lys-207/Lys-325/Lys-347 significantly improved the keratin filament disruption, with K18-R90C/K8-K207R and K18-R90C/K167R having the most prominent effects (Figure 5, A and B). As a control, the 6 individual K8/K18 Lys-to-Arg mutations in the context of WT did not lead to keratin filament disorganization (Supplemental Figure 5). The protective effect of blocking K8 acetylation by Lys mutation, given the similar protective effect of mutating K18 acetylation (in the background of also having K18-R90C), is not surprising due to the obligate heteropolymeric organization of keratins and the human disease phenotype whereby mutation of type I or type II keratins typically behaves similarly (1, 7, 9, 13, 37).

### PN normalizes K14-R125C– and K18-D89H–induced filament disruption and protects from Fas-induced apoptosis.

**Figure 6:**
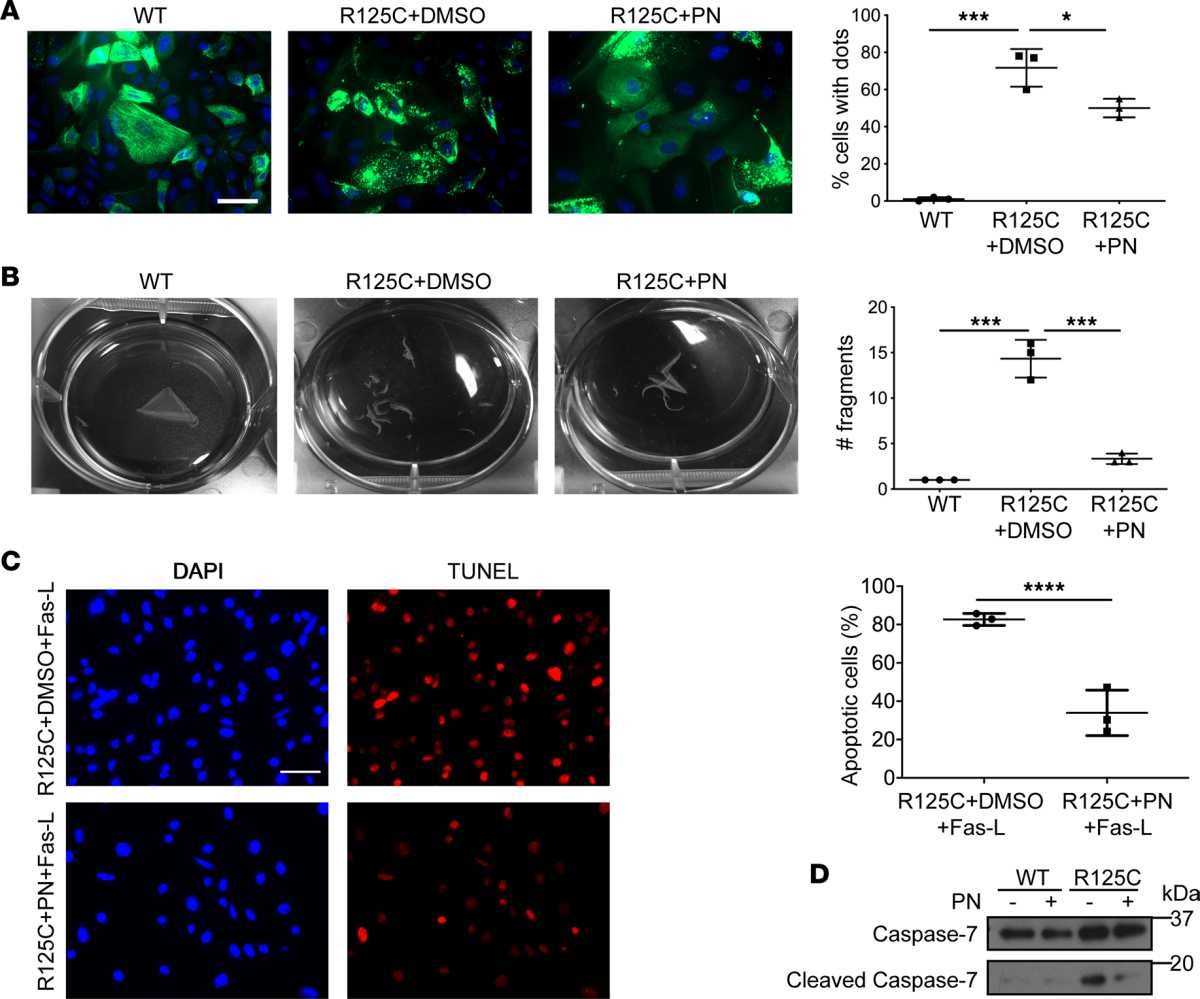
PN normalizes K14-R125C–induced filament disruption and protects against Fas-induced apoptosis and cell response to mechanical stress. (**A**) Type I keratin–deficient keratinocytes were transduced with YFP-tagged K14-WT or K14-R125C. The transduced type I keratin pairs with endogenous type II keratins to form filaments (WT) or dots (R125C). Cells were then treated with vehicle (DMSO) or PN (5 μM, 48 hours). Scale bar: 50 μm. Quantification of the percentage cells with dots/group using pooled counts from 3 separate experiments is shown in the histogram on the right (*P = 0.013, ***P < 0.001). Imaging of the YFP-tagged K14 was done by converting the yellow YFP to a green pseudo color (i.e., from the yellow [570–590 nm] to the green [495–570 nm] wavelength). (**B**) Cells as in panel **A** were treated with dispase to test resistance of the epithelial sheet to mechanical stress. Fragments of the cell sheet were counted and quantified (***P < 0.001). (**C**) Representative images of TUNEL-stained keratinocytes transduced with K14-R125C and then treated with DMSO or PN (5 μM, 48 hours) followed by IFN-γ + Fas-L to induce apoptosis. Quantification of the percentage TUNEL^+^ cells using pooled counts from 3 separate experiments is shown on the right. ****P < 0.001 using unpaired, 2-tailed Student’s *t* test. Scale bar: 50 μm. (**D**) Lysates of cells, similar to those used in panel **C**, were blotted with antibodies against caspase-7 or cleaved caspase-7. For panels **A** and **B**, 1-way ANOVA was used for comparison between treatment groups, and Tukey’s post hoc test was used for 2-group comparisons.

**Figure 7:**
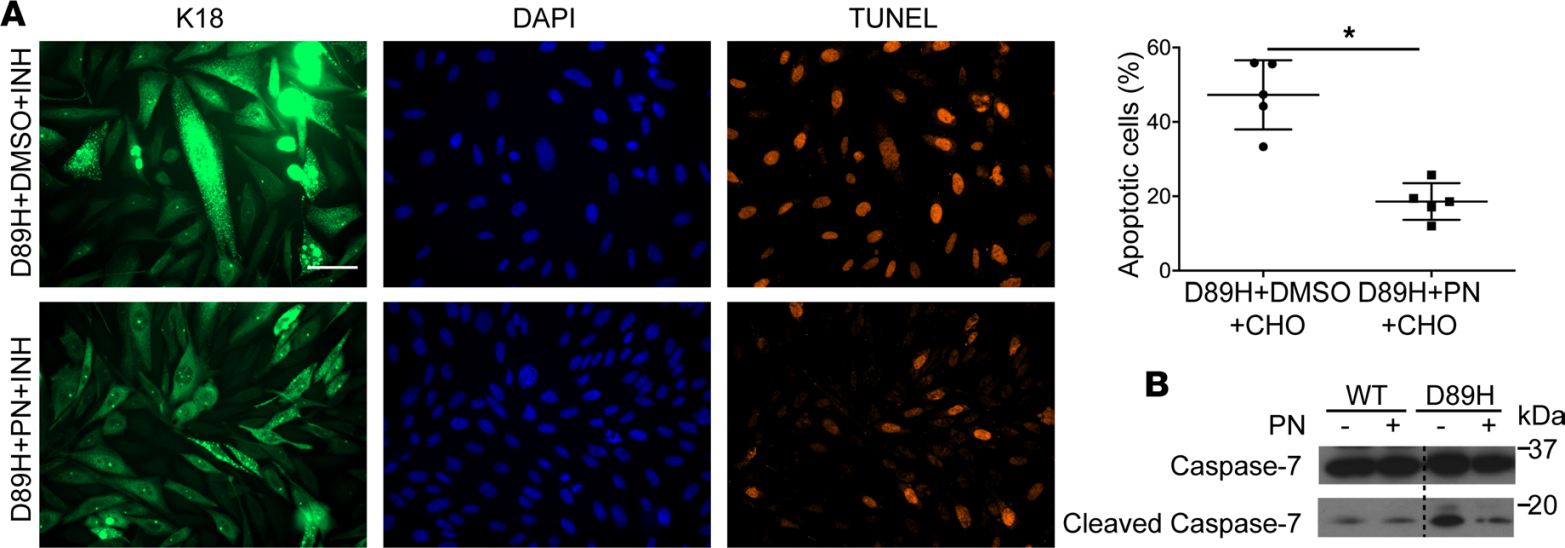
PN normalizes K18-D89H–induced filament disruption and protects against isoniazid-induced apoptosis. (**A**) CHO cells were transduced with GFP-K18-D89H lentivirus then treated with isoniazid (INH; 13 mM) together with DMSO or PN (5 μM, 48 hours). CHO were used instead of A549 cells because they retain the K18-D89H dots, with continued culture better than the A549 cell (not shown). Cells were then fixed and subjected to TUNEL staining. Scale bar: 50 μm. Quantification of the percentage TUNEL^+^ cells using pooled counts from 3 separate experiments is shown on the right (*P < 0.001). Data are presented as mean ± SD. Unpaired, 2-tailed Student’s *t* test was used for comparing the 2 groups. (**B**) Cells (similar to those used in panel **A**) were solubilized and then blotted with antibodies against caspase-7 and cleaved caspase-7. The dotted line indicates that samples in the 2 lanes on the left and right were run in the same gel but are not contiguous.

We also tested the effect of PN in the context of the patient-associated mutations K14-R125C (which causes EBS), and K18-D89H, and K8-K393R (which associate with fatal isoniazid and ezetimibe/simvastatin-related drug-induced liver injury, respectively; ref. 15). We transduced YFP-K14-WT or K14-R125C in keratinocytes deficient in type I keratins (38), followed by treatment with PN or carrier. Importantly, PN cleared the keratinocyte punctate staining significantly (Figure 6A). Moreover, PN increased cell adhesion and resistance to mechanical stress, as determined using a dispase assay (39). As such, an intact epithelial sheet of K14-WT cells lifted off the culture dish in response to mechanical force, while K14-R125C–expressing cells displayed multiple cell-sheet fragments, with PN leading to rescue of the sheet fragmentation (Figure 6B). Furthermore, PN protected K14-R125C cells from IFN-γ– and Fas-L–induced apoptosis, as determined by decreased TUNEL staining and cleaved caspase-7 (Figure 6, C and D). Similarly, PN protected CHO cells transduced with K18-D89H from isoniazid-induced cell apoptosis (Figure 7). The PN protective effect appears to be keratin mutant site-specific since it did not protect cells expressing the K8-K393R variant (Supplemental Figure 6).

## Discussion

In the present study, an unbiased drug screening approach led us to identify PN as a compound with a profound normalizing effect on mutation-triggered keratin filament disruption and consequent protection from apoptosis and injury in cell culture systems and in mice (Figure 1). This finding led us, unexpectedly, to a link between keratin filament organization and the acetylation pathway whereby we demonstrate that K18-R90C mutation leads to keratin hyperacetylation, which is inhibited by PN (Figures 2 and 3). Of note, PN did not protect against apoptosis in a WT keratin background (Figure 1B and Supplemental Figure 1, I–K). We attribute this difference to the selective effect of PN on the hyperacetylated keratin mutant (Figure 2A), which implies that the filament-disrupting keratin mutation is necessary for PN to manifest its protective effect. The importance of keratin deacetylation in normalizing keratin filament was then conclusively demonstrated using pharmacologic means, knockdown of SIRT2, and site-selective mutation of K18 or K8 acetylation in the context of K18-R90C (Figures 2–5 and Supplemental Figures 4 and 5). The importance of sirtuins was further validated by showing that the sirtuin activator NMN has a PN-like effect in terms of keratin filament normalization in cell culture and in vivo (Figure 4). The effect of PN on K18-R90C extends to the K18-D89H variant that is associated with fatal isoniazid-induced liver injury, and to the epidermal K14-R125C mutation that causes the blistering disease EBS (Figures 6 and 7).

Similarly protective effects of PN in liver were observed in other contexts. For example, Cui et al. reported that PN ameliorates fibrogenesis and inflammation in hepatic fibrosis (40). Also, PN exerts beneficial effects on liver injury, lipid metabolism, fibrosis, inflammation, and oxidative stress in mice with metabolic dysfunction–associated fatty liver disease (41). Moreover, PN exhibited protective effects in rodent models of nonalcoholic fatty liver disease and concanavalin A–induced acute hepatitis (42, 43). Although the underlying mechanisms of the previously observed hepatoprotective effects of PN varied in different conditions, most of them were related to the antiinflammatory function of PN. In our study herein, we uncovered that PN protects from genetic keratin-mutation-triggered liver injury by modifying the acetylation of K8/K18, with consequent normalization of keratin filament organization. Although PN appears to have several biologic effects, we link its effect on acetylation, as supported by the findings of SIRT2 knockdown and pharmacologic intervention.

Among all IF posttranslational modifications, phosphorylation is the best studied, with keratin hyperphosphorylation being a hallmark that accompanies essentially every form of epithelial cell stress (22). In that context, the multikinase inhibitor PKC412 had a similar effect to that of PN in normalizing K18-R90C dots (17), although PN manifested an earlier beneficial effect (Supplemental Figure 1C). However, enzyme activators typically offer several advantages as compared with inhibitors, such as having greater target specificity (44). Also, while some double K8 but not K18 phospho (Ser-to-Ala)/R90C mutants led to conversion of keratin dots to filaments, triple acetyl-phospho-R90C mutation of K8/K18 had no added benefit in normalizing keratin filaments, and some phospho/combined mutants resulted in more dots (Supplemental Figure 7). This suggests that keratin deacetylation may be upstream of phosphorylation, or that keratin deacetylation may have a more dominant effect compared with hypophosphorylation. More importantly, PKC412 acts by dephosphorylating myosin rather than major K8/K18 phosphorylation sites (17), and PKC412 does not lead to K18 deacetylation (not shown).

PN has also been studied as an NF-κB inhibitor (45). PN was characterized as an NF-κB inhibitor that can directly inhibit the p65 subunit and has been used as an NF-κB inhibitor in several studies (46, 47), aside from other finding describing its role in acetylation (28, 29). Of note, SIRT2 interacts with NF-κB cytoplasmic p65 and deacetylates p65 at Lys-310, leading to its inactivation (48). Our findings herein provide another link between PN and SIRT2.

The role of sirtuin pathways and acetylation has been reported previously in hepatic pathophysiology. For example, hepatic SIRT2 protein expression was inversely correlated with alcoholic liver disease development (49). Moreover, SIRT1-mediated deacetylation regulates hepatic lipid metabolism and controls the regenerative response of liver (50, 51). In our study herein, the link of deacetylation to mutant keratin filament normalization was based on several lines of evidence, including point mutations of keratin acetylation, pharmacologic inhibition and activation of SIRT2, and knockdown of SIRT2. We examined keratin acetylation using an anti–acetylated lysine antibody, but the 3 antibodies we tested from different vendors show varied patterns (Supplemental Figure 8), which suggested that these antibodies have different acetylation site recognition patterns. For example, for some of the antibodies, PN in some cases led to decreased antibody reactivity (deacetylation), while other proteins appeared to undergo hyperacetylation after PN treatment. This indicates that PN’s effect on acetylation varies, depending on the protein. These findings also suggest that PN may not be a pan-inhibitor or pan-activator of acetylation, which explains the seemingly different PN inhibitory effect on HDAC1 and -4 (29, 30) but activation of SIRT2.

The insolubility of PN limits its clinical utility, but its water-soluble prodrug DMAPT has PN-like biologic effects and might prove to be useful clinically (52). Our findings showed that DMAPT has similar normalizing effects on mutant keratin filament disorganization in cell culture and in mouse liver as compared with PN (Supplemental Figure 1, D and H). PN is known to have pleiotropic effects, including antiinflammatory, antioxidative, and antifibrotic effects, as well as linkage with multiple signaling pathways, including NF-κB, p53, HIPPO, TLR4, and STAT3 (40–43, 45). PN also destabilizes microtubules by forming tubulin adducts on cysteine and histidine residues (53). We hypothesize that compounds targeting SIRT2 directly (e.g., NMN) are more likely to be clinically applicable for the keratin mutations we studied, and offer an exciting potential particularly given that several sirtuin-activating compounds are presently undergoing clinical trials (54).

**Figure 8:**
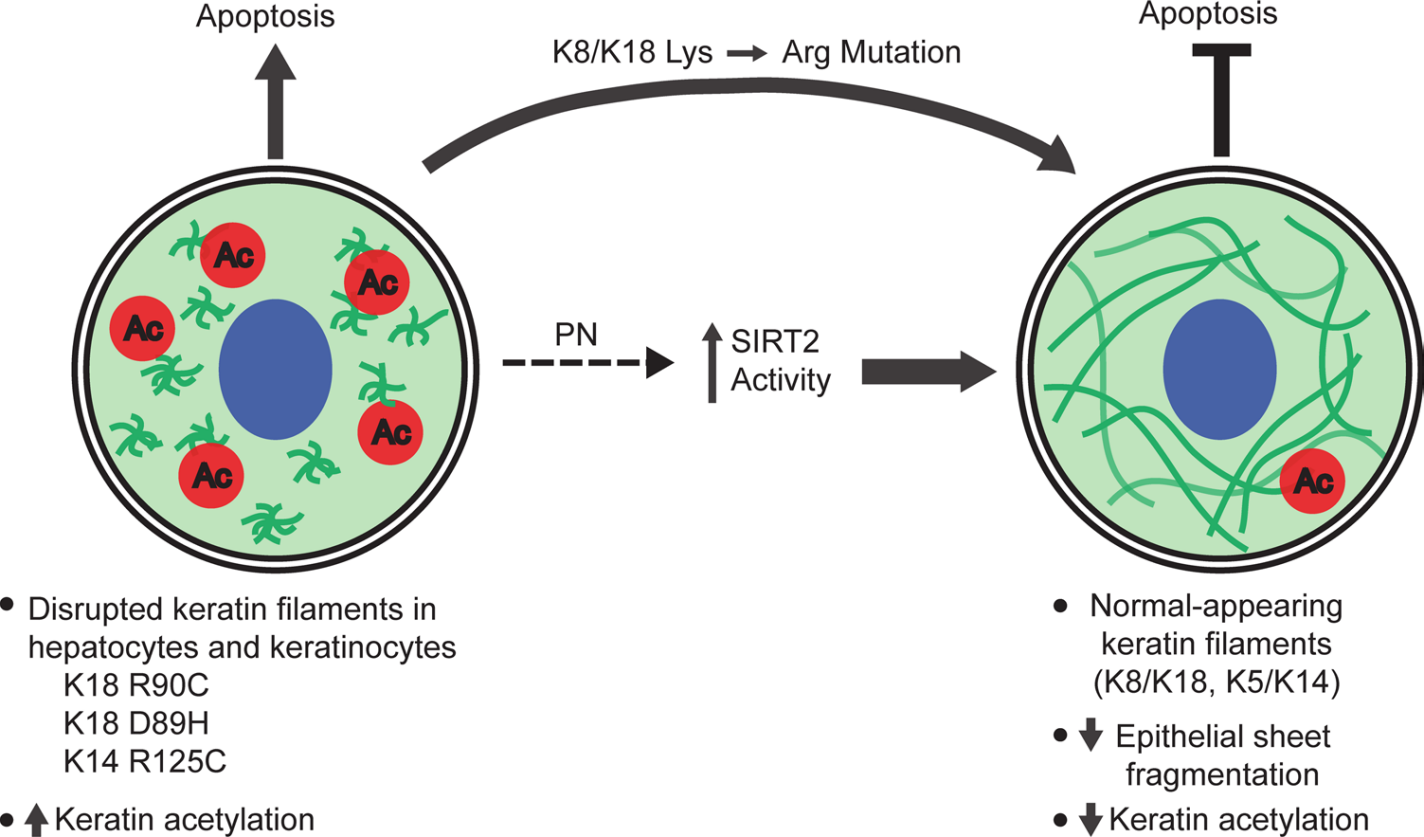
Summary of the effect of PN via SIRT2 activation on mutant keratin filament organization and susceptibility to apoptosis. The schematic summarizes our overall findings. Ac, acetylation; PN, parthenolide; the wavy green lines represent keratin filaments that are aggregated before PN treatment that become extended filaments after treatment with PN.

In conclusion, the targeting of simple-epithelial K18 and epidermal K14 acetylation, in the context of K18 and K14 mutations that have dramatic clinical consequences (fatal drug-induced liver injury/blistering skin disease) and cell biologic manifestations (morphologic change from filaments to dots with the biologic consequence of IF aggregation and predisposition to apoptosis), offers potential therapeutic options and a mechanism for regulating keratin filament organization (Figure 8). One potential benefit of identifying uniquely functioning drugs that may have clinical utility in keratin- and other IF-related disorders is that they may offer the opportunity to be used, after testing experimentally, as combination drugs. This applies to the demonstrated protective effect in keratin mutants by 2 separate Ser/Thr or Tyr kinase inhibitors (17–19) and the observation herein by targeting protein acetylation. Such an approach is likely to be particularly beneficial for drugs that are already in clinical use and, therefore, can be repurposed for keratin- and other IF-related disorders. Such drug combinations may offer several potential advantages, including decreased toxicity profiles (if effective at lower doses when combined) and having an additive or even synergistic effect.

## Methods

### Mouse studies.

The following mouse lines were used: nontransgenic FVB/N (Jackson Labs), transgenic mice that express human K18-WT or K18-R90C (FVB/N) (12), and K18^–/–^ mice (FVB/N) (14). For the Fas experiments, mice (16–18 weeks, sex matched) were injected intraperitoneally (i.p.) with PN (Sigma-Aldrich; 1 mg/kg) or vehicle (DMSO) daily for 4 days. On day 4 after treatment, liver injury was induced by i.p. injection of Fas-L (0.15 μg/g body weight). For NMN treatment, mice were administered NMN (Sigma-Aldrich; 500 mg/kg body weight) i.p. daily for 4 days (prepared in PBS, pH 7.4). Animals were euthanized by CO_2_ inhalation 5 hours after Fas-L administration. Histological analysis to assess the extent of hemorrhage involved using a 0 to 5 scoring system without any knowledge by the scorer of the experimental arms: 0, no hemorrhage; 1, less than 20% of the section area covered by hemorrhage; 2, 20%–40% hemorrhage; 3, 41%–60% hemorrhage; 4, 61%–80% hemorrhage; and 5, greater than 80% hemorrhage.

### Histology and immunofluorescent staining.

Hematoxylin and eosin staining was performed by the Microscopy and Image Analysis Core at the University of Michigan. Frozen mouse liver sections or cells were prepared for immunofluorescent staining as described previously (55). Slides were fixed in acetone (10 minutes, –20°C), air dried for 20 minutes, and incubated in blocking solution (PBS, 2% bovine serum albumin, 2% goat serum). Primary antibodies (e.g., anti-keratin antibody L2A1, which recognizes human K18; ref. 12; see Supplemental Table 1 for a list of additional antibodies that were used in this study) were added (22°C, 1 hour) followed by 3 rinses with PBS and then addition of the secondary antibodies conjugated to Alexa Fluor 488 or Alexa Fluor 594 (Life Technologies). Staining was visualized using an Olympus FluoView-500 laser scanning confocal microscope and a 60x water-immersion (1.2 NA) objective and FluoView software (version 5.0; Olympus). For quantification, 10–20 fields were used for each treatment condition, and at least 3 individual experiments were repeated. Data are presented as mean ± SD of these individual experiments.

### High-throughput screening.

The screening strategy and procedure was as described in detail previously (16). Briefly, A549 cells (human lung carcinoma cell line, obtained from the American Type Culture Collection) were transduced with GFP-tagged K18-WT or K18-R90C, and K8-WT lentiviruses. This cell line was selected because it is nearly 100% transduced and it forms readily discernable extended filaments or dots, depending on the expression of WT or mutant keratins, respectively. Both K8 (type II keratin) and K18 (type I keratin) need to be present in order to form filaments, otherwise expression of a single type I or type II keratin in cells results in rapid degradation due to lack of stabilization by the partner keratin (56). After 1 day of transduction, cells were seeded into 384-well plates for screening, followed by addition of the compounds (Center for Chemical Genomics, University of Michigan) on day 2 after transduction. A total of 1,037 compounds were tested that include drugs that target the epigenetic, autophagic, redox, protease, kinase, Wnt, and cannabinoid pathways. After 2 days in the presence of the test compounds, cells were fixed for 20 minutes using 4% paraformaldehyde in PBS and then counterstained with 100 mg/mL 4′,6-diamidino-2-phenylindole (DAPI; Invitrogen) in PBS. Images were acquired using the Image Xpress Micro (IXM) XLS High Content Imaging System (Molecular Devices), followed by analysis using MetaXpress software (Molecular Devices). A total of 24 compounds were selected from the initial screening (performed in 384-well plates) for validation using chamber slides, with transduced A549 cells treated with the 24 compounds. From these compounds, PP2 (19) and PN had the most prominent effect in terms of decreasing the extent of K18-R90C filament collapse and dot formation. Further validation was then carried out by testing the dose response for PN (data not shown). For PN, the optimal dose for cell culture studies (in terms of maximal effect on normalizing the keratin filaments and having limited toxicity) was 5 μM. The stock solution of PN was 50 mM (dissolved in DMSO).

### Cell culture, apoptosis induction, and construct transfection.

CHO (Chinese hamster ovary), BHK (baby hamster kidney), and NIH/3T3 (mouse fibroblast) cells were obtained from the American Type Culture Collection and maintained as recommended by the supplier. NIH/3T3 cells were used when a system that lacks endogenous keratins was desired (they provide well-extended filaments). When sufficient protein expression was needed, BHK cells were used (which also lack keratins), although they do not provide the typical well-extended filaments (12, 55). CHO cells were used for the K18-D89H and K8-K393R experiments since the typical keratin dot pattern with these constructs tended to revert to normal filaments in A549 cells after 3–4 days of transduction (data not shown). Mouse type I keratin–deficient keratinocytes were obtained from type I keratin–null embryos (38) followed by establishment of the cell line as described previously (57). To test susceptibility to apoptosis, GFP-K18-WT– and GFP-K18-R90C–transduced A549 cells were pretreated with DMSO or PN (5 mM, 48 hours) followed by addition of IFN-γ (R&D Systems; 40 ng/mL, 6 hours) and then Fas-L (CH11, Millipore; 100 ng/mL, 24 hours). Lipofectamine 2000 (Invitrogen) was used for plasmid transfection and Lipofectamine RNAiMAX (Invitrogen) was used for siRNA transfection as recommended by the manufacturer. Plasmids encoding FLAG-tagged HDAC6 and FLAG-tagged SIRT2 were obtained from Addgene. SIRT2 siRNA was purchased from Origene. Cells were harvested for biochemical and immunofluorescent staining 48 hours after transfection.

### Preparation of cell and tissue lysates, immunoprecipitation, and immunoblotting.

Cells were harvested and solubilized in SDS-containing sample buffer with 5% β-mercaptoethanol to obtain total cell/tissue homogenates. Cultured cells or liver tissues were homogenized in buffer containing 1% NP-40 for SIRT2 immunoprecipitation, or 1% Empigen buffer for acetyl-lysine immunoprecipitation. The solubilization buffers were supplemented with the protease inhibitors 50 mM NaF, 0.1 μM okadaic acid, 0.5 μM trichostatin A (TSA), and 10 mM nicotinamide (NAM).

Immunoprecipitation was performed with antibodies against SIRT2 or AcK conjugated to Dynabeads Protein A (Invitrogen) (3 hours, 4°C). For detection of the acetylated proteins, we initially tested 3 separate anti-AcK antibodies (from Abcam, EMD Millipore, and Cell Signaling Technology) by immunoblotting using A549 cells transduced with GFP-K18-R90C and then cultured in the presence or absence of PN. Each of the antibodies gave a different profile, with the Abcam antibody providing the strongest reactivity with the acetylated keratin species (Supplemental Figure 8), so it was selected for use in all subsequent experiments. Immunoblotting was carried out after transferring proteins to polyvinylidene difluoride membranes. Visualization of the antibody-reactive species was done using enhanced chemiluminescence. Immunoprecipitation and immunoblotting were repeated at least 3 times and band intensity was analyzed using ImageJ software (NIH).

### SIRT2 activity assay.

The assay was performed according to the instructions of the human SIRT2 Direct Fluorescent Screening Assay Kit (Cayman Chemical). In general, the reaction plates included 100% initial activity wells with human SIRT2 recombinant; test wells with PN (0, 2.5–50 μM), NMN (activator control; 5 μM and 50 μM), NAM (inhibitor control; 50 μM); and background wells (without the addition of PN/NMN/NMA) that included only the reaction buffer. Each concentration was tested in triplicate and the fluorescence of each well was detected by an excitation wavelength of 350–360 nm and an emission wavelength of 450–465 nm. The average fluorescence was determined from the triplicate wells in each group, and SIRT2 activity was calculated using the formula, SIRT2 activation (%) = ([sample average fluorescence minus background average fluorescence] minus [initial average fluorescence minus background average fluorescence] ÷ [initial average fluorescence minus background average fluorescence]) × 100.

### Dispase assay.

Keratinocytes were seeded in 6-well plates. High calcium (2 mM) medium was added to the confluent cell layers for 24 hours, followed by washing 3 times with PBS. Dispase (STEMCELL Technologies) was then added (2.4 U/mL) and incubated at 37°C for 30 minutes. The plates were then subjected to orbital rotation (150 rpm, 30 minutes, 37°C) and fragments were counted.

### Lentiviral constructs and site-directed mutagenesis.

All the WT and mutants constructs with or without GFP tagging (K18-WT, K8-WT, K14-WT, K18-R90C, K18-D89H, K8-K393R, K14-R125C) were generated using the QuikChange Site-Directed Mutagenesis Kit (Agilent Technologies). The constructs were confirmed by DNA sequencing. Viral vectors were prepared with assistance of the Vector Core at the University of Michigan.

### Statistics.

Statistical analysis was performed using Prism 6 software (GraphPad Software). Statistical comparisons were done using the unpaired *t* test or, for samples with 3 or more groups, by 1-way ANOVA followed by Tukey’s post hoc test.

### Study approval.

All studies and procedures involving mice were approved by the University of Michigan and Rutgers University Institutional Animal Care and Use Committees and in accordance with the NIH *Guide for the Care and Use of Laboratory Animals* (National Academies Press, 2011).

## Author contributions

JS, HG, LR, TMM, LL, and MBO designed the experiments. JS, PL, HG, and LL performed the experiments. All authors provided experimental input, interpreted the data, and approved the manuscript. JS, PL, HG, and MBO wrote the manuscript and prepared the figures with input by DBL, KR, and QX.

## Supplementary Material

Supplemental data

## Version 1 07/24/2023

Electronic publication
