# Supplementary material for: Deacetylation via SIRT2 prevents keratin-mutation-associated injury and keratin aggregation
Source: JCI Insight. 2023 Jul 24;8(14):e166314. doi: 10.1172/jci.insight.166314 (PMC10443796; doi:10.1172/jci.insight.166314)
Supplement: Supplemental data [file jciinsight-8-166314-s189.pdf]

Supplementary Figures

Supplementary Figure 1. PN and DMAPT normalize K18-R90C filament disorganization and protect against Fas-L induced injury in cells and mice. (A) K18-R90C-expressing A549 cells were treated with DMSO, PN (5μM), PKC412 (0.8μM) or PN (5μM) + PKC412 (0.8μM) for 48h. The percent cells with dots was then determined (*p<0.05, **p<0.01, ***p<0.001). **(B)** A549 cells were transduced with lentivirus expressing GFP-K18-R90C together with K8. Cells were treated with DMSO or PN (5μM, 48h). High salt extraction was performed as described in the Methods section to enrich for the keratin fraction. The high salt extract was analyzed by SDS-PAGE and Coomassie staining (lower panel) or blotted with antibodies to the indicated keratins. Note that PN does not alter the expression of several tested keratins. **(C)** GFP-K18-R90C expressing cells were treated with DMSO, PN (5μM) or PKC412 (0.8μM) for the indicated times then fixed and stained with DAPI. Higher doses of PKC412 were toxic (not shown). The percent cells with dots was then determined for each time point. Findings were pooled from three independent experiments (***p<0.001, ****p<0.0001). **(D)** GFP-K18-R90C expressing cells were treated with DMSO or DMAPT (5μM) for 2d. Quantification of the percent cells with dots from 3 independent experiments is shown (**p<0.01). **(E)** Quantification of the percentage of apoptotic cells in Fig.1C (****p<0.0001). **(F)** Transgenic mice that express human K18-R90C were treated with DMSO or PN (5 mice/group; 1mg/kg mouse weight/d) for 4d. The percent hepatocytes with dots is shown, with representative images in Fig.1D. (***p<0.001). **(G)** Liver homogenates were prepared then blotted with antibodies to K8 and K18. Note that keratin levels did not change after PN treatment. A Coomassie stain is included as a loading control. **(H)** K18-R90C expressing transgenic mice were fed with DMSO or DMAPT (5 mice/group; 1mg/kg mouse weight/d) for 4d. The percent hepatocytes with dots is shown (**p<0.01). **(I-K)** Non-transgenic FVB/N mice, human K18-WT-expressing mice (as a control for the human K18-R90C-

expressing mice), and K18-null mice (which lack cytoplasmic intermediate filaments because of the instability of K8 when expressed alone) were treated with DMSO or PN as in panel F then challenged with Fas-L. All mice were in the FVB/N genetic background. Sera were collected followed by measurement of ALT. Error bars represent mean ± SD. For panels A and C, one-way ANOVA was used for comparison between treatment groups, and Tukey post-hoc test was used for two-group comparisons. For panels D-F, H-K, unpaired, 2-tailed Student's t test was used for two-group comparisons.

Supplementary Figure 2. PN alters K18 acetylation via SIRT2 but not HDAC6 or the other sirtuins. (A) BHK (selected for their high transfection efficiency that provides sufficient material for biochemical analysis) and A549 cells were co-transfected with K18-R90C and K8, together with flag-SIRT2 or flag-HDAC6 followed by high salt extraction after 2d. The extracts were blotted with anti-AcK antibody. Coomassie stain of the extracts is included as a loading control. **(B-H)** mRNA levels of *SIRT1-7* were tested by qRT-PCR in GFP-K18-WT/GFP-K18-R90C-transduced A549 cells treated with DMSO or PN (5μM, 48h). **(I)** Lysates from GFP-K18-WT/GFP-K18-R90C-transduced A549 cells were blotted with antibodies to SIRT2 or SIRT5. **(J)** Colocalization coefficient of K18 and SIRT2 in Fig.2E (**p<0.01 unpaired, 2-tailed Student's t test).

Supplementary Figure 3. PN alters K18 solubility in cells and mice. (A) Livers from mice that express K18-R90C (treated with DMSO or PN for 4d) were subjected to serial fractionation (with pelleting after each fraction collection) using hypotonic detergent-free buffer, then 1% NP-40, then 1% Empigen. The final 'Pellet' corresponds to the post-Empigen solubilization pellet. The fractions were then blotted with antibodies to K18 and SIRT2. Coomassie staining of the fractions is included as a loading control. Three independent livers from each group (DMSO or PN) were subjected to the

serial fractionation. **(B)** Ac-α-tubulin was determined by immunoblotting of liver lysates from mice given DMSO/PN. Coomassie staining is included as a loading control. n=4 (DMSO group), n=5 (PN group).

Supplementary Figure 4. Pharmacologic inhibition or knockdown of SIRT2 enhances K18 acetylation and blocks the keratin filament normalizing effect of PN. (A) Quantification of the percent cells with dots in Fig.3A (*$p<0.05$, **$p<0.01$). **(B)** Quantification of the percent cells with dots in Fig.3C (**$p<0.01$). **(C)** K18-R90C-expressing cells were cultured with control or SIRT5 siRNA (24h) then treated with DMSO/PN (48h). The percent cells with dots is shown. **(D)** Duplicate cells from panel C were collected and acetylated proteins were immunoprecipitated using anti-AcK antibody followed by blotting with anti-K18 antibody. Coomassie staining is included to show equal loading. For panels A-C, one-way ANOVA was used for comparison between treatment groups, and Turkey post-hoc test was used for two-group comparisons.

Supplementary Figure 5. Effect of K8/K18 Lys-to-Arg mutations on keratin filament organization. Representative images of NIH-3T3 cells expressing the indicated K8 (WT or the K207R/K325R/K347R acetylation mutants) and K18 (WT or the K131R/K167R/K214R acetylation mutants) constructs. Scale bar=50μm.

Supplementary Figure 6. PN has no effect on normalizing K8-K339R induced filament disruption and protecting against simvastatin-induced apoptosis. (A) CHO cells were transduced with GFP-WT-K18 together with K8-K393R lentiviruses and treated with simvastatin (1μM) together with DMSO or PN (48h). Simvastatin was selected because it was the drug that resulted in fatal liver injury in the patient who harbored the K8-K393R variant. The cells were fixed then subjected to TUNEL staining. The percentage of TUNEL^{+} cells is shown, and for this variant PN did not protect from apoptosis and did not improve the number of cells with dots. Scale

bar=50µm. **(B)** Lysates from cells similar to those used in panel A (+simvastatin, transduced with WT K8 or K8 K393R) were analyzed by immunoblotting with apoptosis markers.

Supplementary Figure 7. The effect of phospho mutations on keratin filament organization. (A) A549 cells were co-transduced with lentiviruses that include: (i) Upper row: K18-R90C plus K8 [WT or one of the three phospho-mutants: S24A, S74A, S432A], (ii) Middle row: K18-R90C or the K18-R90C/K8-K207R double mutant plus the indicated K8 acetyl or phospho-mutants, and (iii) Bottom row: K8-WT plus K18-R90C that also carries combinations of the acetylation and phosphorylation mutants. Bar=50µm. **(B)** Quantification of the extent of cells with dots from panel A. Arrows highlight the transductions that resulted in an increase in the number of cells with dots as compared with K18-R90C alone [i.e., the K18 constructs: (i) phospho-mutant R90C/S53A, and (ii) Phospho/Ac-mutant R90C/S34A/K131R], (*p<0.05, **p<0.01, ***p<0.001). Error bars represent mean ±SD. ANOVA was used for measurement between treatment groups, and t-test was used for two-group comparisons.

Supplementary Figure 8. Anti-acetylated lysine antibodies from different commercial vendors showed varied patterns of acetylation site recognition. A549 cells were transduced with lentivirus expressing GFP-K18-R90C together with K8. Cells were treated with DMSO or PN (5µM, 48h). Total lysates were analyzed by SDS-PAGE followed by immunoblotting with three anti-acetylated lysine antibodies from the indicated vendors (Abcam; EMD, EMD Millipore; CST, Cell Signaling Technology). Coomassie staining (right panel) is included as the loading control.

Supplementary Table 1. Commercially purchased antibodies used in the study.

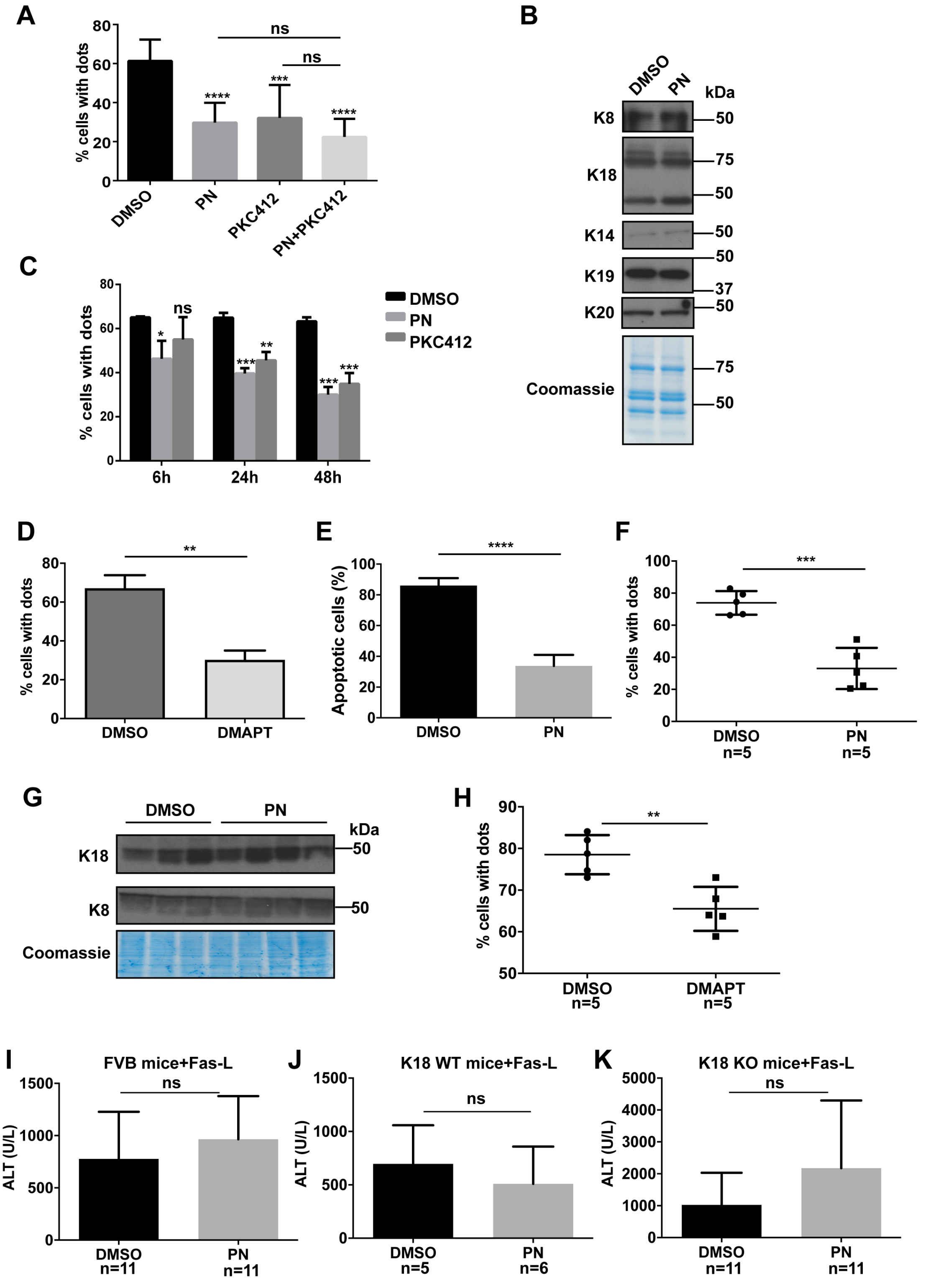

Supplementary Figure 1

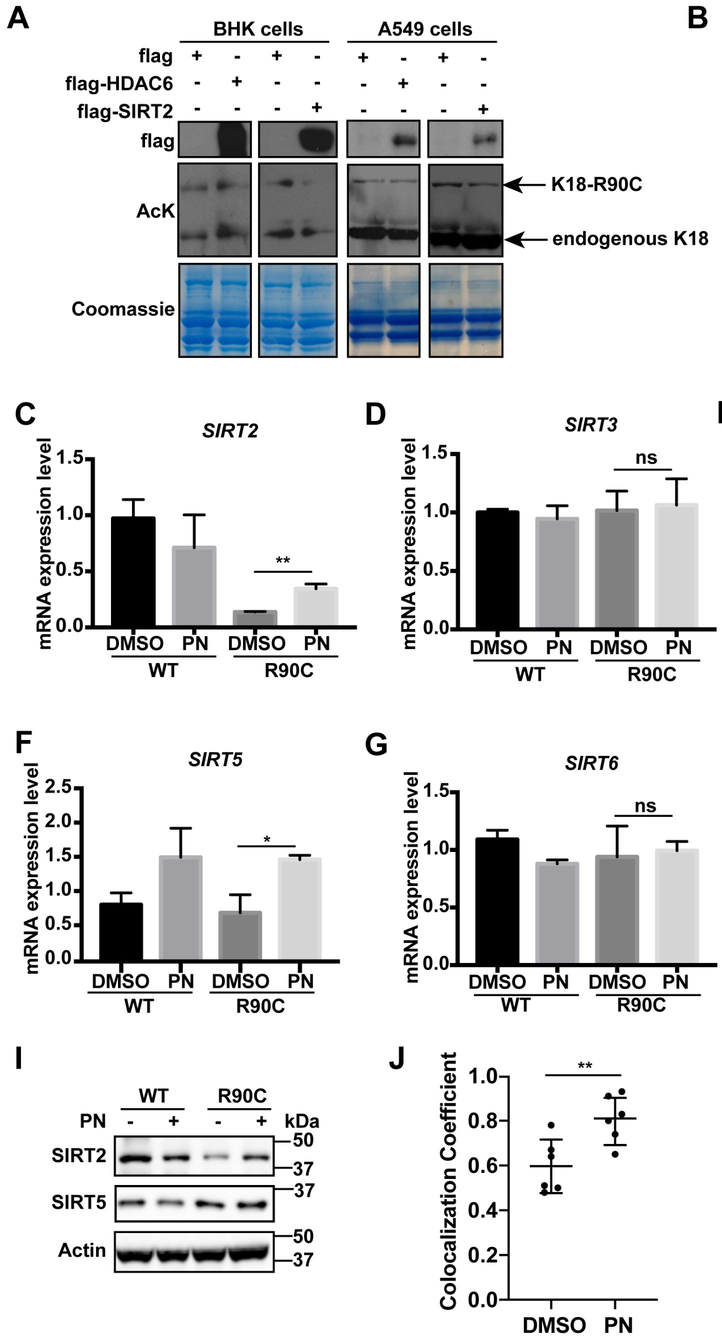

Supplementary Figure 2

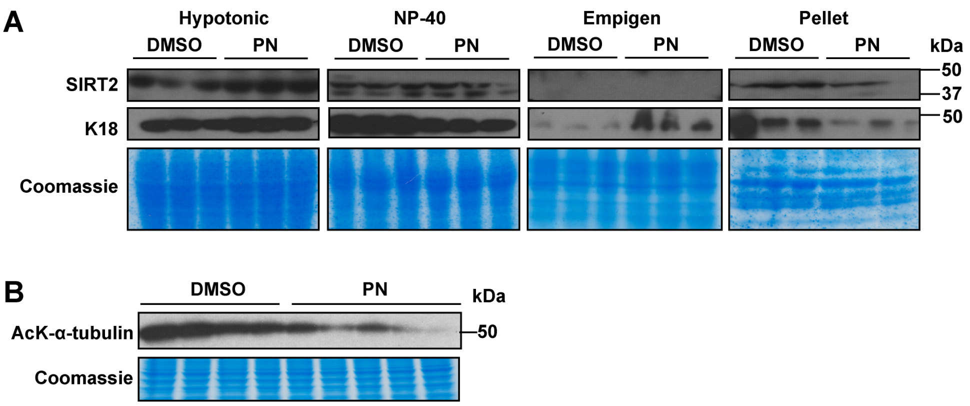

Supplementary Figure 3

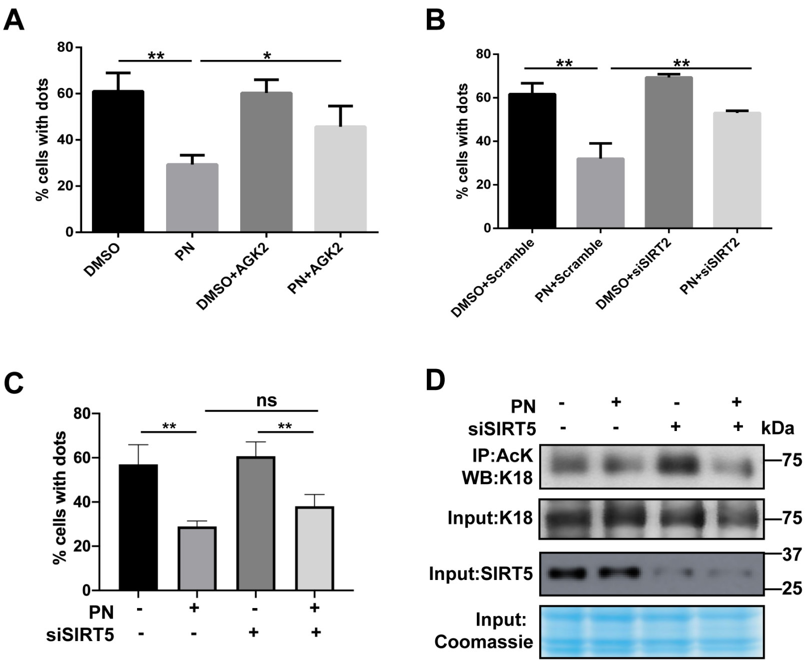

Supplementary Figure 4

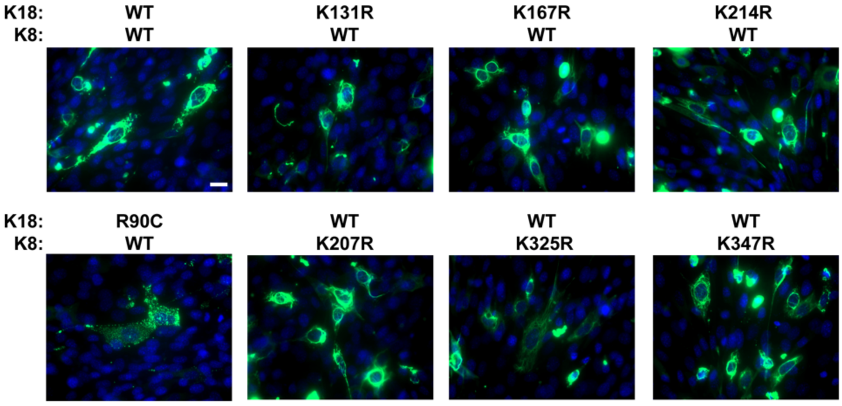

Supplementary Figure 5

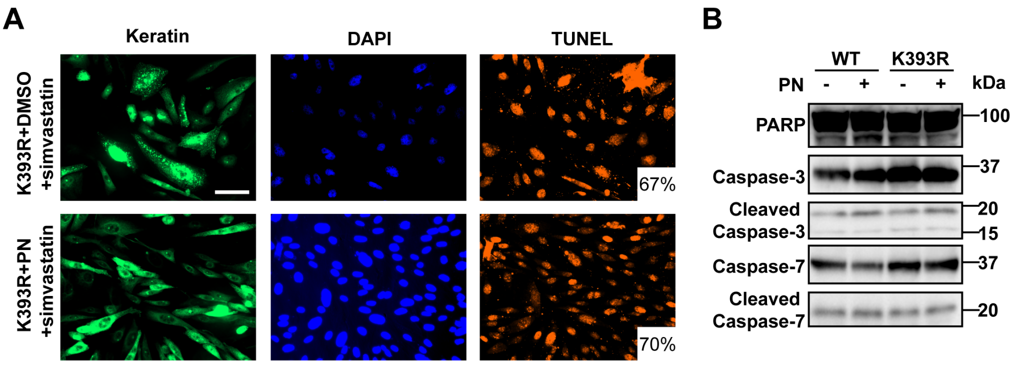

Supplementary Figure 6

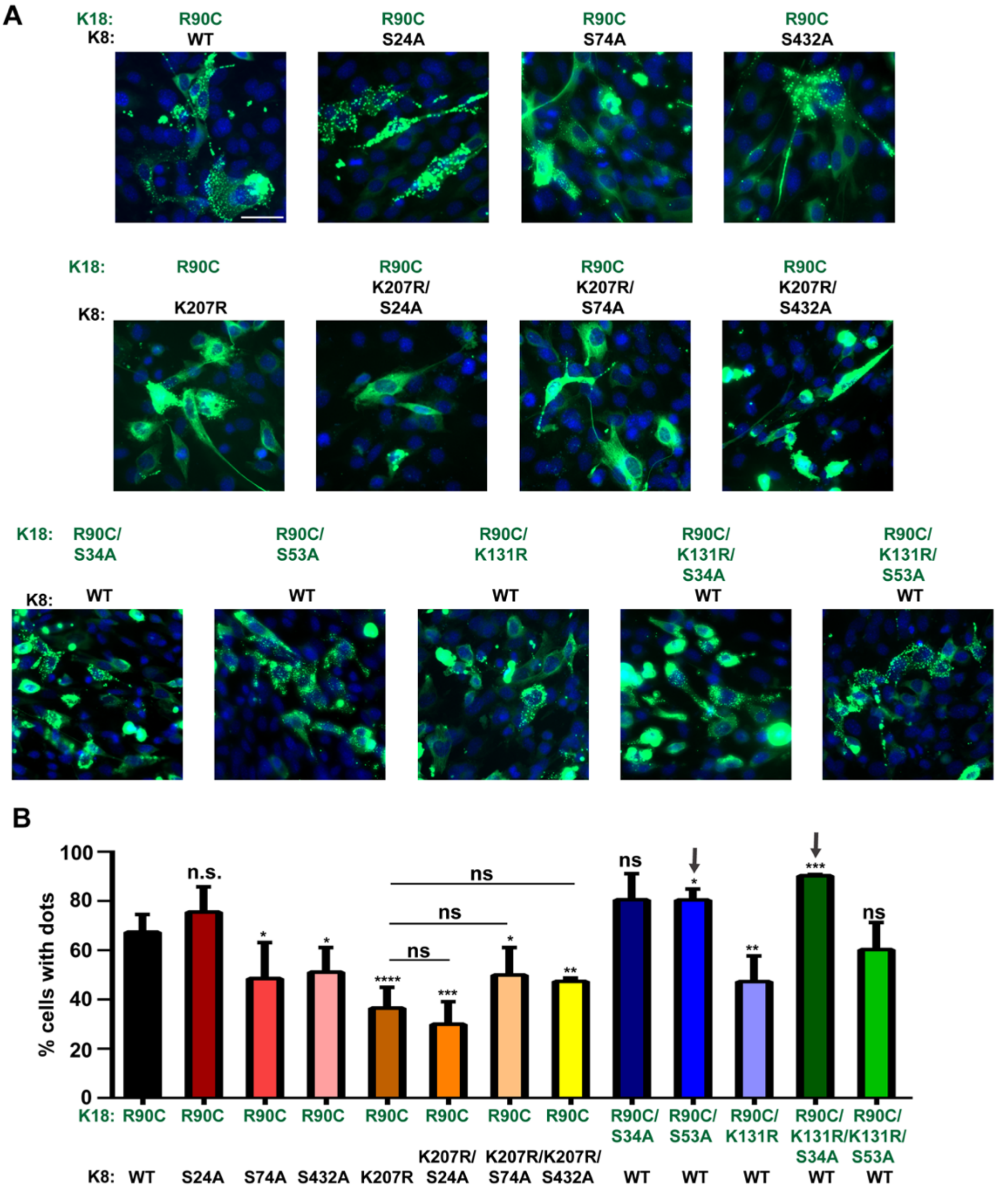

Supplementary Figure 7

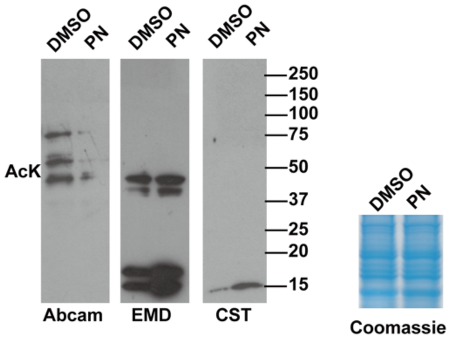

Supplementary Figure 8

Supplementary Table 1. Commercially purchased antibodies used in the study.

Antibody	Manufacturer	Catalogue
PARP (46D11)	Cell Signaling Technology	9532
Caspase-3	Cell Signaling Technology	9662
Cleaved Caspase-3 (Asp175) (5A1	Cell Signaling Technology	9664
Caspase-7	Cell Signaling Technology	9492
Cleaved Caspase-7 (Asp198)	Cell Signaling Technology	9491
Acetyl-lysine (Ac-K-103)	Cell Signaling Technology	9681
Acetyl-lysine	Abcam	ab80178
Acetyl-lysine	EMD Millipore	ST1027
SIRT2	Abcam	Ab67299
AcK-α-tubulin (Lys40) (D20G3)	Cell Signaling Technology	5335
HSP70	ThermoFisher Scientific	MS-482-P
Keratin 8 (TS1)	ThermoFisher Scientific	MS -997-P
Keratin 18 (DC10)	ThermoFisher Scientific	MS-142-P
Keratin 14	ThermoFisher Scientific	RB-9020-P
Keratin 19 (A53-B/A2.26)	ThermoFisher Scientific	MS-198-P
Keratin 20 (K20-3)	ThermoFisher Scientific	61032
Tubulin alpha (DM1A)	ThermoFisher Scientific	MS-581-P
Tubulin beta (TBN06)	ThermoFisher Scientific	MS-1226-P
F-Actin (NH3)	ThermoFisher Scientific	MA1-80729
